# OmicsOne: associate omics data with phenotypes in one-click

**DOI:** 10.1186/s12014-021-09334-w

**Published:** 2021-12-11

**Authors:** Hui Zhang, Minghui Ao, Arianna Boja, Michael Schnaubelt, Yingwei Hu

**Affiliations:** 1 School of Medicine, Johns Hopkins University, Baltimore, MD 21287 USA; 2 Mount Hebron High School, Ellicott City, MD 21042 USA

**Keywords:** Proteomics, Glycoproteomics, Phenotype association, Bioinformatics, Software, Ovarian cancer

## Abstract

**Background:**

The rapid advancements of high throughput “omics” technologies have brought a massive amount of data to process during and after experiments. Multi-omic analysis facilitates a deeper interrogation of a dataset and the discovery of interesting genes, proteins, lipids, glycans, metabolites, or pathways related to the corresponding phenotypes in a study. Many individual software tools have been developed for data analysis and visualization. However, it still lacks an efficient way to investigate the phenotypes with multiple omics data. Here, we present OmicsOne as an interactive web-based framework for rapid phenotype association analysis of multi-omic data by integrating quality control, statistical analysis, and interactive data visualization on ‘one-click’.

**Materials and methods:**

OmicsOne was applied on the previously published proteomic and glycoproteomic data sets of high-grade serous ovarian carcinoma (HGSOC) and the published proteome data set of lung squamous cell carcinoma (LSCC) to confirm its performance. The data was analyzed through six main functional modules implemented in OmicsOne: (1) phenotype profiling, (2) data preprocessing and quality control, (3) knowledge annotation, (4) phenotype associated features discovery, (5) correlation and regression model analysis for phenotype association analysis on individual features, and (6) enrichment analysis for phenotype association analysis on interested feature sets.

**Results:**

We developed an integrated software solution, OmicsOne, for the phenotype association analysis on multi-omics data sets. The application of OmicsOne on the public data set of ovarian cancer data showed that the software could confirm the previous observations consistently and discover new evidence for HNRNPU and a glycopeptide of HYOU1 as potential biomarkers for HGSOC data sets. The performance of OmicsOne was further demonstrated in the Tumor and NAT comparison study on the proteome data set of LSCC.

**Conclusions:**

OmicsOne can effectively simplify data analysis and reveal the significant associations between phenotypes and potential biomarkers, including genes, proteins, and glycopeptides, in minutes to assist users to understand aberrant biological processes.

**Supplementary Information:**

The online version contains supplementary material available at 10.1186/s12014-021-09334-w.

## Background

A phenotype can be defined as any observable characteristic or state of an organism resulting from interactions between genes, environment, disease, molecular mechanisms, and chance [1]. The purpose of phenotype association analysis in genomics and proteomics for disease studies is to illustrate the relationship between protein expression and clinical phenotypes. With the advancements of high-throughput “omics” technologies, including genomics, epigenomics, transcriptomics, proteomics, protein modifications, glycomics, lipidomics, and metabolomics, the incredible volume of data has been produced [2–7]. Predictably, the trend of generating large datasets will continue as novel technologies are being developed and current approaches advance. In this era of omics data explosion, an automated solution for multi-omics phenotype association analysis will significantly increase knowledge discovery from a large amount of data in the studies of diseases, such as cancers.

In the past decades, many efforts have been made in bioinformatics tools development for automated omics data analysis and visualization, including commercial solutions of Ingenuity Pathway Analysis [8] (Ingenuity Systems, QIAGEN Inc.) and ProteinCenter (Thermo Scientific/Proxeon) and non-commercial tools, including infernoRDN (former DanTE and DanteR) [9, 10], ProteoSign [11, 12], GproX [13], DAPAR/ProStaR [14], GiaPronto [15], Perseus [16], PANDA-view [17], and IOAT [18]. Those tools were developed to perform the statistical analysis of quantitative discovery proteomics experiments, which contain procedures to do data processing, perform null hypothesis significance tests, generate the visualization of quantitative proteomics data and other -omics data, and the following Gene Ontology (GO) [19] enrichment analysis. However, all these tools are designed for routine workflow of data analysis for omics data. There are still some problems with automated phenotype association analysis. (1) Those tools lack a practical integration mode. The functional modules are separated in different pages and not optimally organized for an efficient automated pipeline. (2) Those tools lack the support of investigation of phenotypes and association analysis between phenotype and post-translational modifications (PTMs), especially for glycosylation. (3) Most of these tools only provide limited extensibility for customized databases and scripts and static data visualization.

To address these issues, here we present the tool OmicsOne, a software developed in Python based on Dash framework [20] that can perform the automated phenotype association analysis for multi- “omics” data in a ‘one-click’ mode. The quantitative expression matrices and clinical information table were the only required inputs for initializing phenotype of association analysis. The results are reported in tab-separated text.txt or.csv file formats and visualized in an interactive web-based graphical interface in a web browser by a simple ‘one-click’ button. In addition, OmicsOne added supports for annotation and phenotype association analysis for intact glycopeptides. Protein post-translational modifications (PTMs) play a crucial role in protein and gene expression and various cellular mechanisms, increasing the complexity and diversity of the proteome [21–23]. Protein glycosylation is one of the most abundant examples of PTMs [24], as it is a critical factor in various biological functions such as cell–cell recognition, cell–cell adhesion, determining protein structure, and involvement in human disease [25–29]. Because of its link to human disease, glycosylation research has allowed a link to be established between altered glycoproteins and abundant cancer cell traits [30]. All the functional modules of OmicsOne support the analysis of eligible expression matrices of mRNA, protein, and intact glycopeptides to discover interesting molecules or pathways related to the corresponding phenotypes in a study. OmicsOne also supports interactive data visualization and extensibility to integrate with users’ customized Python scripts and databases in the data processing pipeline, facilitates a deeper interrogation of a dataset. OmicsOne is free available on GitHub (https://github.com/huizhanglab-jhu/OmicsOne) and can be installed and run locally in Python 3.8 environment in Microsoft Windows. The minimum hardware configuration requirements are 2-cores CPU (e.g., Intel i5-6300U) and 12 GB RAM.

## Methods

### Input file format

**Fig. 1:**
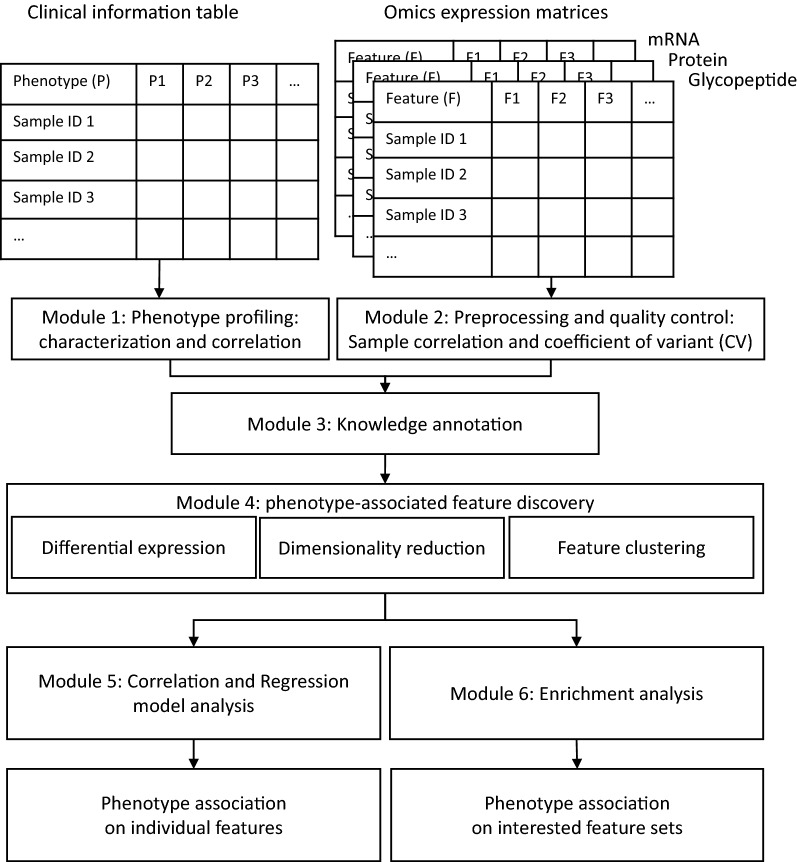
The software operation process. Module 1: Phenotype characterization and correlation; Module 2: Data quality evaluation; Module 3: Knowledge database annotation for all identifications; Module 4: Feature (gene, protein, PTM) selection using differential expression analysis, dimensionality reduction, and clustering; Module 5: Correlation and regression model analysis for phenotype associated with the individual feature; Module 6: Enrichment analysis for phenotype association with feature sets

OmicsOne was initially designed for isobarically labeled quantitative proteomics data (e.g., tandem-mass-tag (TMT)) but can find applications in label-free quantitation and Data Independent Acquisition (DIA) datasets, as well as other “omics” data if the data fits the input format shown in Fig. 1. The two sample data sets are included in the installation package and installed with the software together. In the default settings, OmicsOne accepts the log2-transformed expression matrices saved as ‘wide’ format, in which samples names are the row indices and feature names (gene name or glycopeptide) are the column labels (Fig. 1). To be compatible with intact glycopeptide analysis, the name of intact glycopeptide, also called glycoform, is defined as GeneName_PeptideStartSite_PeptideSequence_GlycositeNumber_GlycositePosition_Glycancomposition. The name of protein is the corresponding gene name.

The sample data sets embedded in OmicsOne installation are also downloadable in the Github repository. OmicsOne also allows users to add their customized annotation databases in the sample folder for knowledge annotation, pathway databases for enrichment analysis.

### Software modules

We developed OmicsOne under Python 3.8 for automated multi-omics data analysis to discover molecular changes and pathways associated with phenotypes. OmicsOne integrated scientific Python packages for statistical calculation and data visualization, including NumPy(v1.21.4) [32], SciPy(v1.7.1) [33] for statistical calculation, Pandas(v1.3.4) [34] for data table manipulation, Scikit-learn(v0.24.2) [35] for machine learning, GSEApy(v0.10.5) [36–38] for gene set enrichment analysis, and Plotly(v5.3.1) and Dash(v2.0.0) [20] for interactive data visualization and dashboard construction. All functions were integrated into an efficient analysis suite after modular development, which provides six main functional modules: (1) phenotype profiling, (2) data preprocessing and quality control, (3) knowledge annotation, (4) phenotype associated features discovery, (5) correlation and regression model analysis for phenotype association analysis on individual features, and (6) enrichment analysis for phenotype association analysis on interested feature sets (Fig. 1). The results were visualized as webpage-based interactive figures. The details of the six implemented modules are described in the following sections.

### Module 1. Phenotype profiling

Understanding the composition of data cohort is always the first and critical step for all the following studies for phenotype association analysis. OmicsOne supports statistics on the phenotype information to calculate the population of samples in different phenotype groups. OmicsOne will also investigate the pairwise correlation of phenotypes to reveal the dependencies between the phenotypes.

### Module 2. DATA preprocessing and quality control

It is often necessary to preprocess the raw data before data analysis to fit the algorithm requirements and control data quality. OmicsOne provides several essential preprocessing functions, including (1) Log-transformation algorithm, which supports the conversion of the expression values to log2 values. OmicsOne accepts log2-transformed data by default. (2) Normalization algorithm. We implemented the commonly applied median normalization method to adjust the median values of all features in all samples to the same (default is 0) to reduce the potential batch effect and measurement erros. (3) Noise filtration algorithm. We removed the features expressed less than 50% (user defined) samples as noise features, and (4) Imputation algorithm. Three basic imputation methods were implemented in OmicsOne including: *GlobalMin*: impute the missing value using a scaled global minimum value; *SampleMin*: impute scaled minimum value in the row (the minimum value of all features in this sample); and *FeatureMin*: impute scaled minimum value in the row (the minimum value of this feature among all samples).

The evaluation of the reproducibility of quality control samples is another critical step before the phenotype association analysis. OmicsOne supports calculating the correlation values of technical or biological replicates and coefficient of variation (CV) of the selected quality control samples to estimate the reproducibility of measured gene or protein level expression.

### Module 3. Knowledge annotation

The gene annotation function can help the understanding of biological functions. A quick annotation tool is critical for automated data analysis and manual investigation. In OmicsOne, the features are automatically annotated and linked to the knowledge databases (e.g., UniProtKB [39] for gene and protein annotation and N-Glycositeatlas [40] database for N-linked glycosite annotation). Up to our knowledge, there is not a large-scale database containing the specific information to link glycosites to phenotypes of diseases. Thus, this tool is useful to link the results of phenotype association analysis of glycopeptides directly to the knowledge database. N-GlycositeAtlas is a database containing sample information of historically published glycosites. OmicsOne provides the function for both database annotation based on GlycositeAtlas and phenotype association analysis for the newly discovered glycopeptides. Users can also add their customized database to extend the annotation or export their identification with original GlycositeAtlas for future studies.

### Module 4. Phenotype-associated feature discovery

OmicsOne provides three sub-modules for phenotype-associated feature discovery, including differential expression analysis, dimensionality reduction (also called decomposition), and feature clustering.

Differential expression analysis is a method delineating altered expression profiles of features, such as genes, proteins, and PTMs, which offers the greatest insight into aberrant biology in comparative studies (e.g., Tumor vs. Non-tumor). The algorithms of hypothesis tests (e.g., t-test and Wilcoxon) implemented in OmicsOne can identify the significant, differentially expressed features, leveraging multiple statistical tests for paired or independent groups. The student t-test is the most commonly used statistical hypothesis test in which the test statistic follows a Student’s t-distribution. Wilcoxon rank-sum test is a non-parametric statistical hypothesis test used to compare the locations of two independent populations respectively [41]. For dependent groups, OmicsOne supports the corresponding paired t-test and Wilcoxon signed-rank test for the comparison. The results can be directly visualized in the interactive volcano plot for exploring all the features involved in the tests. Under the default settings, OmicsOne reports the features as significantly altered features if there are more than 1.5 fold change and a less than 0.01 adjusted p-value (adjusted by Benjamini–Hochberg Procedure [42]) between the two compared groups. The intermediate testing result will be stored as a.csv file and provides candidate features for regression and enrichment analysis.

The dimensionality reduction method is a valuable and common approach to classify samples based on the most prominent factors driving different phenotypes without prior knowledge, especially for samples with thousands of features. Among a series of dimensionality reduction methods, Principal component analysis (PCA) [43] is one of the most widespread methods implemented in OmicsOne supported by Python package: Scikit-learn [35], to separate samples and identify the signature gene groups associated with the corresponding sample groups. The top 10 most prominent features can be visualized in each principal component. The most prominent features (default is 100) based on the contribution score were selected for phenotype association analysis. The contribution score is defined as $\sum_{i=1}^{n}V{R}_{i}*\frac{{abs(V}_{ij})}{\sum_{j=1}^{m}abs({V}_{ij})}$, where $m$ features are decomposed by $n$ principal components (PCs), $V{R}_{i}$ is the explained variance ratio of $P{C}_{i}$, ${V}_{ij}$ is the variance of feature $j$ contributes to $P{C}_{i}$.

Feature clustering is based on the hierarchical clustering supported by Python package Scipy [33] to find gene sets sharing similar alteration patterns in different phenotypes. The expression values of each feature were z-score transformed crossing samples before clustering. User can define the cluster number. The clustered gene sets respective to each phenotype are exported for the following analysis.

### Module 5. Correlation and regression model analysis for phenotype association analysis on individual features

OmicsOne provides phenotype association analysis for individual features. The features involved in the gene sets obtained from the differential expression analysis, dimensionality reduction, and feature clustering methods can be investigated individually for phenotype association. The correlation analysis and logistic regression analysis are provided for individual features associated with categorical phenotypes. The features having a correlation p-value < 0.05 are considered as phenotype-associated features. The logistic regression model applied on the phenotype and feature expression is helpful to justify if an individual feature can be considered as a potential indicator for the phenotype prediction.

### Module 6 enrichment analysis for phenotype association analysis on interested feature sets

The gene sets can be further investigated by the subsequent enrichment analysis, over-representation analysis (ORA) using GSEApy [36–38] to discover pathways enriched behind genes associated with different phenotype states. GSEApy is a python implementation for gene set enrichment analysis (GSEA) and wrapper for Enrichr [36–38]. OmicsOne can automatically recall functions of GSEApy to do enrichment analysis on the selected significant features from the upstream analysis to reveal the pathways and biological functions involved by these features.

### Interactive data visualization of results

OmicsOne reports intermediate and finalized results in tables (.csv or.txt) and the corresponding interactive figures for all data analysis. The interactive figures are generated using Plotly in Dash framework for direct checking. OmicsOne automatically generates intermediate tables in.csv or.txt (Tab-separated) file for phenotype association results for each step of processing.

## Results

The public proteomic data sets of high-grade serous ovarian carcinoma (HGSOC) [44] and lung squamous cell carcinoma (LSCC) [45] were applied to demonstrate the functions of OmicsOne. The clinical information table of phenotypes was exported to ‘wide’ format files (support tab-separated.txt file or Excel file), in which sample names are the row indices, and phenotype names are column labels. The tag of ‘(Categorical)’ or ‘(Numerical)’ was added in each column label of phenotype for OmicsOne to recognize the data types of phenotypes.

**Fig. 2:**
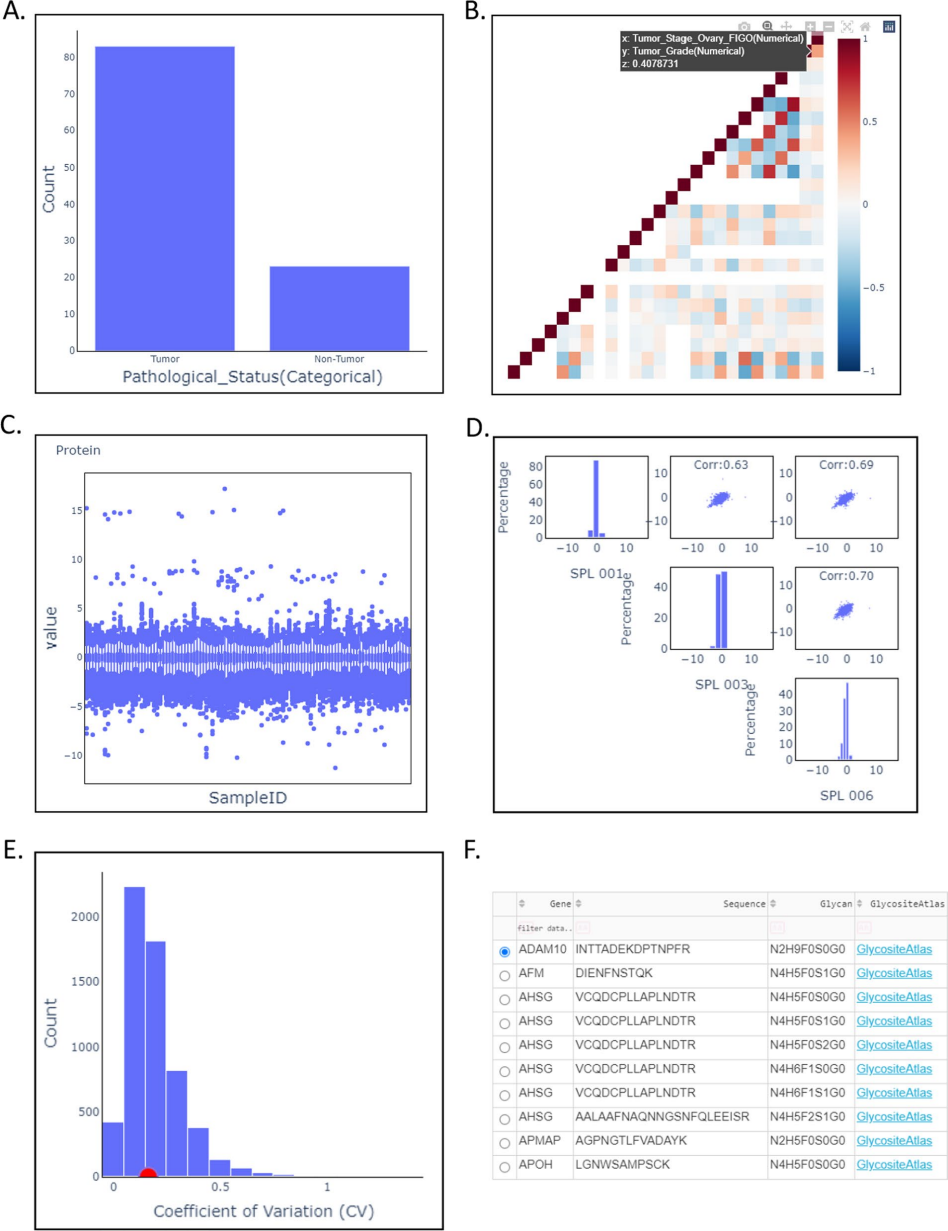
The software operation process (including phenotype profiling, processing, data quality evaluation, and database annotation) taking the proteome and glycoproteome data of HGSOC as an example. **A** Phenotype profiling by interactive characterization of the population in different phenotypes. **B** Phenotype profiling by phenotype correlation for revealing dependencies among different phenotypes. **C** Box plots of expression values of all normalized samples in the proteome data set of HGSOC. **D** Data quality evaluation by correlation of QC samples in the proteome data set of HGSOC. **E** Data quality evaluation by a distribution of coefficient of variant (CV) values of features in three samples in the proteome data set of HGSOC. **F** Table of feature details of intact glycopeptide identified in the glycoproteome data set of HGSOC associated with linkage to the knowledge database of *N*-GlycositeAtlas

OmicsOne was firstly applied on the public proteomic and glycoproteomic data set in the Additional tables of HGSOC [44] to demonstrate the functions. The results are shown in the Additional file 2: Table S1 for proteomic data analysis and Additional file 2: Table S2 for glycoproteomic data analysis. The phenotype table contains 106 samples (83 tumors and 23 non-tumors from normal fallopian tubes) associated with 9 classes of phenotypes (such as pathological status, tumor cellularity, and tumor grade.) and 3 sample clusters information. The sample clustering results were treated as categorical phenotypes in this study. The characterization of phenotype pathological status of the tumor and non-tumor samples was shown in Fig. 2A to demonstrate module 1 of phenotype profiling. The categorical phenotypes were automatically converted to numerical phenotypes to perform correlation analysis, as shown in Fig. 2B. We found that the Tumor_Stage_Ovary_FICO is positively correlated to the Tumor_Grade score (0.4) as expected. The phenotype correlation table also reveals other phenotype dependency information we may need to consider in the following investigation.

This investigation involved two expression matrices of protein and intact glycopeptides, including 5916 proteins and 365 intact glycopeptides, respectively. In this study, we regarded proteins or intact glycopeptides as features describing the samples. These features described each sample in a high-dimensional space. Although OmicsOne provides the preprocessing functions in module 2 of data preprocessing and quality control, it also accepts data preprocessed using different preprocessing methods outside. The expression matrices of protein and intact glycopeptides have been log2-transformed, normalized, and have no missing values. The expression distribution in each sample is shown in Fig. 2C. The quality control module calculates the correlation of samples and coefficient of variant of features crossing samples to evaluate the variances of samples. The protein expression matrix of three samples (‘SPL 001’,’SPL 003’, and ‘SPL 006’) from sample cluster 1 were selected to demonstrate the functions. As shown in Fig. 2D and E, we observed that the average correlation is 0.67 and median CV is 0.16, demonstrating that the evaluation procedure can provide valid information for quality control. In module 3 of knowledge database annotation, two knowledge databases, UniProtKB [39] and *N*-Glycositeatlas [40], were provided to annotate the identifications of proteins and intact glycopeptides. The annotation table supports quick query of target features, linkage to the database for further knowledge discovery, and feature selection for the following phenotype-feature association analysis (Fig. 2F).

**Fig. 3:**
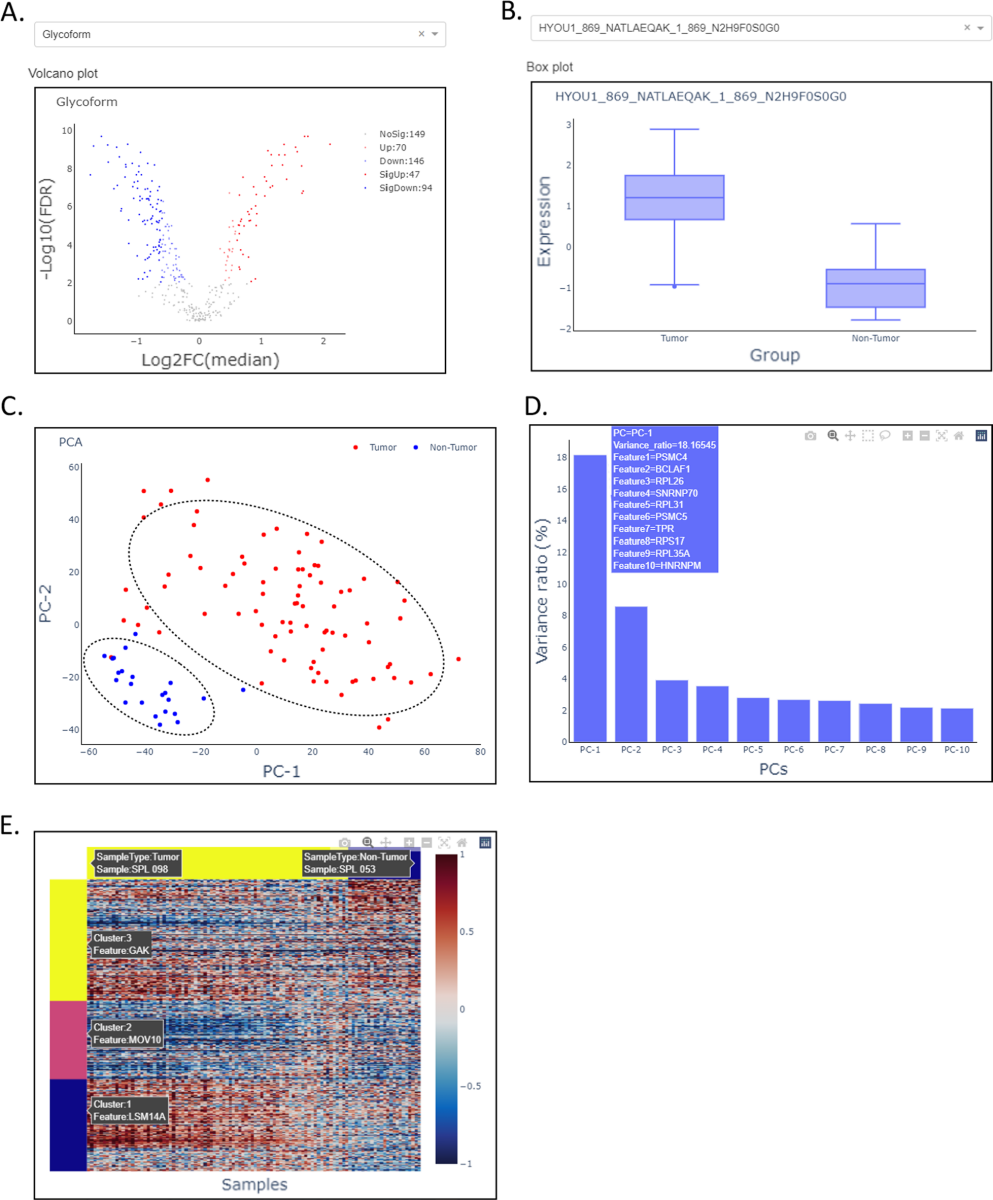
The phenotype-associated feature discovery procedures (including differential expression analysis, Dimensionality reduction, and feature clustering). **A** Interactive volcano plot of the result of differential expression analysis using hypothesis tests and multiple tests corrections applied on the glycoproteome data of HGSOC. **B** Interactive box plot for each feature (glycopeptide) expressed in different phenotypes (e.g., Tumor vs. Non-Tumor samples) in the glycoproteome data set of HGSOC. **C** Dimensionality reduction using principal component analysis (PCA) for most variant features in the proteome data set of HGSOC. **D** Variance ratio values of top 10 principal components (PCs) used in the PCA model applied on the proteome data set of HGSOC. The top 10 features contributed to each PC are provided in the hover data information. **E** Clustering analysis for features identified in the proteome data set of HGSOC associated with the phenotype of pathological status (Tumor and Non-tumor)

In the module 4 of phenotype-associated feature discovery, we implemented three functions: differential expression analysis, dimensionality reduction, and feature clustering. The purpose of this module is to find individual features or feature sets relevant to specific phenotypes. There are 47 significantly up-regulated and 94 down-regulated intact N-linked glycopeptides were discovered in tumor samples compared with non-tumor samples using Wilcoxon rank-sum tests and considering Benjamini-Hochberg (BH) adjusted p-value < 0.01 and fold change > 1.5 (Fig. 3A). The box plot of individual feature HYOU1_869_NATLAEQAK_1_869_N2H9 in different phenotypes of tumor and non-tumor samples was visualized as shown in Fig. 3B. In the section of dimensionality reduction, OmicsOne showed that the tumor and non-tumor samples were basically classified using their protein expression data (Fig. 3C) and listed the PCs sorted descending by their explained variance ratio as well as the top 10 most prominent features contributed to the PCs (Fig. 3D). In the feature clustering section, users can intuitively view the effect of features clustered under different phenotypes (Fig. 3E) and select the suitable cluster number to get feature sets for the following enrichment analysis to find the pathways behind these clusters of features. After this procedure, we can collect interesting features from the three upstream analysis methods, including up-or down-regulated features in differential expression analysis, top contributed features in PCs to explain the sample variances, and clustered feature sets relevant to phenotypes.

**Fig. 4:**
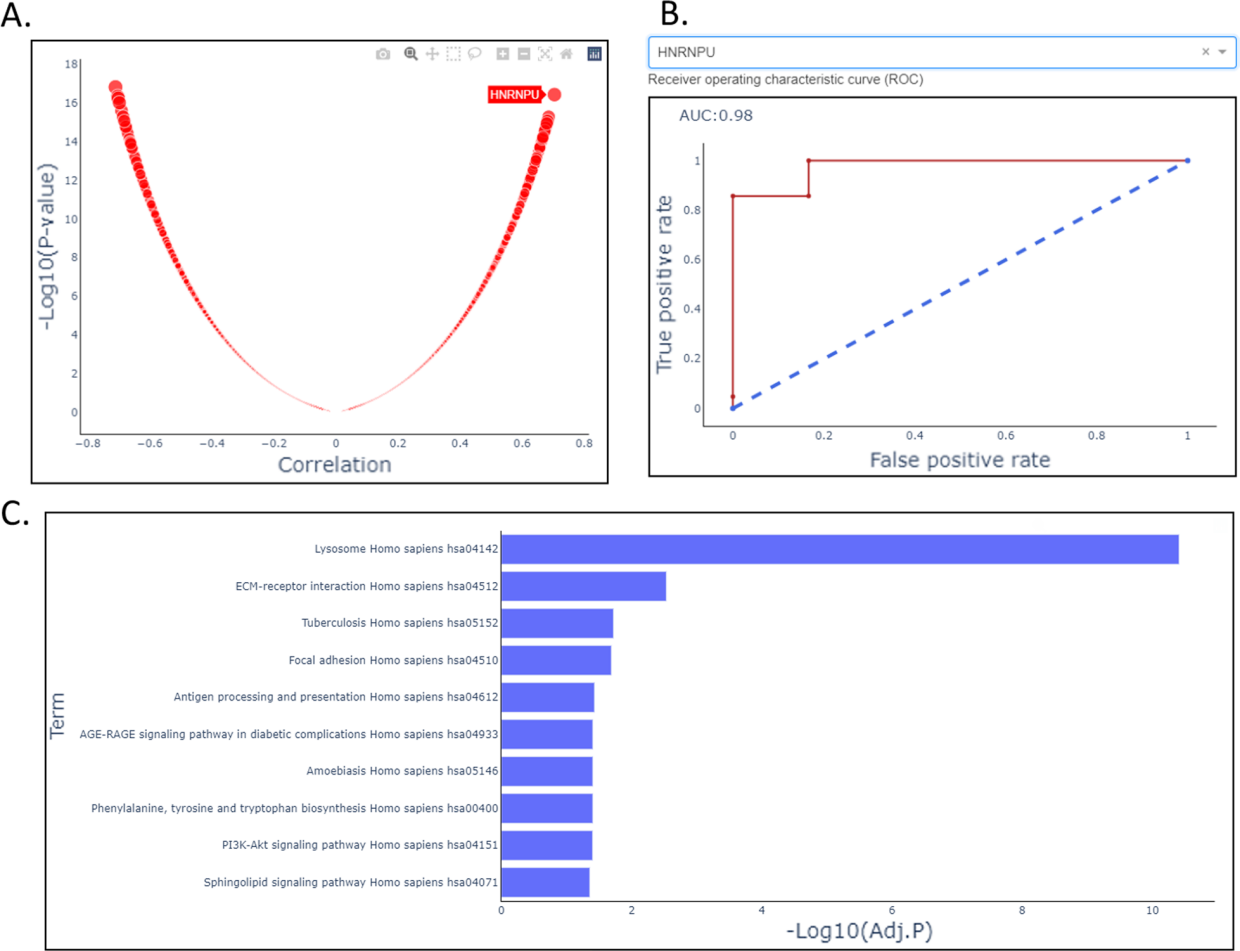
The modules of phenotype association analysis, including phenotype associations with individual features and phenotype association with enriched pathways, applied on the proteome and glycoproteome data sets of HGSOC. **A** Correlation between phenotype ‘Tumor’ of pathological status and the protein expression of gene HNRNPU in all samples using the proteome data set of HGSOC. **B** Receiver operating characteristic (ROC) curve for regression model between Pathological Status (Tumor) ~ protein expression (HNRNPU). **C** The enriched KEGG (2016) pathways identified by Over-representation analysis (ORA) on the gene list of significantly up-regulated glycopeptides

OmicsOne provides the functional module of correlation and regression model analysis (module 5) for the investigation of phenotype and individual feature association. As shown in Fig. 4A, we found that the protein HNRNPU is the most positively correlated with the pathological status of Tumor (Fig. 4A), and the logistic regression result showed that the area under the receiver operating characteristic curve (ROC) is 0.98 (Fig. 4B). The module 6 of enrichment analysis provides an over-representation method for discovering pathways enriched by the interesting gene sets. For example, the lysosome pathway is enriched in genes of the significantly up-regulated intact glycopeptides identified in the differential expression analysis section (Figs. 3A and 4C). This observation is consistent with the result in the previous publication [44].

OmicsOne was also applied on the proteome data set from LSCC to confirm its performance [44]. The clinical information table and proteome expression able were extracted from the original Additional file 2: Table S1, Additional file 4: Table S3 respectively. The PCA result (Additional file 1: Fig. S1A) was consistent with the original observation, in which the Tumor and NAT samples were separated clearly [44]. The differential expression analysis of the 99 paired Tumor and NAT samples was executed in OmicsOne using the nearly same settings according to the method section in the publication of LSCC to find the significant tumor-associated proteins (FDR < 0.01 and fold change > 2). The result was also consistent with the original observation (Additional file 1: Fig. S1B). We applied two databases of Gene Ontology (GO) biological process (BP) (v.2021) [46] and MSigDB_HallMark (v.2020) [36] for the enrichment analysis. We consistently observed the pathways related to Cell Proliferation and DNA Repair enriched in the significantly up-regulated proteins in tumors, while the pathways related to Cell Adhesion and Acute Immune Response enriched in the significantly down-regulated proteins in tumors (Additional file 4: Table S3).

## Discussion

OmicsOne is an efficient automated tool to associate the alteration of features with phenotypes. The software uses empirical settings to build a robust working pipeline for standard association analyses in ‘one-click’ mode and allows the interactive manipulation for tuning the analysis to fit the customized requirement. The ‘one-click’ mode can speed up the discovery of interesting features and feature sets and the following phenotype association analysis. However, we still strongly suggest that users carefully investigate each module's settings and results and not use OmicsOne as a black box. Thus, we developed a webpage-based dashboard in OmicsOne, which integrates interactive data visualization of results and the corresponding parameter settings to make the analysis clearer and more efficient to validate. Users can monitor the results of each module in real-time during the running of the whole data analysis.

OmicsOne supports phenotype profiling, knowledge annotation, and intact glycopeptide analysis. It provides a convenient way to associate intact glycopeptide to clinical phenotypes (Fig. 3A and B). The literature information of the intact glycopeptide can be easily accessed via the linkage in the annotation table. OmicsOne also provides intuitive and interactive data visualization for the analysis results. Users can directly select the interesting data points in the figure to obtain detailed information for further investigation. In this demonstration investigation, we reported two observations in the protein and glycopeptide data sets of HGSOC. In the protein data set, the protein HNRNPU is the most positively correlated with the pathological status of Tumor (Fig. 4A), and the corresponding area under the ROC curve (AUC) score is 0.98 using a logistic regression model for tumor prediction. The median fold change of HNRNPU in protein expression is 1.67 and adjusted p-value < 0.01 in the differential expression analysis result of comparing tumor and non-tumor samples. These tests can be efficiently accomplished in OmicsOne in minutes and suggest that HNRNPU may be a potential biomarker for HGSOC, supported by the recent studies [44, 47]. Moreover, we observed a glycoform of NATLAEQAK with oligomannose glycan N2H9, of which the gene HYOU1 was recently reported as a promotor for cell growth and metastasis via activating PI3K/AKT signaling in epithelial ovarian cancer and predicts poor prognosis [48]. It would be interesting to investigate the role of glycosylation in this promotion mechanism.

The performance of OmicsOne was further demonstrated by the application on the proteome data of LSCC. The results of PCA and differential expression analysis for the comparison between Tumor and NAT samples (Additional file 1: Fig. S1A and S1B) confirmed the reproducibility of the previous observations. OmicsOne also provided more details of the analysis results. The enriched pathways in the tumor-associated proteins (Additional file 4: Table S3) showed classical histological features, including the upregulation of Oxidative phosphorylation and Glycolysis related pathways and downregulation of immune response. The enriched EMT pathway in the new subtype ‘EMT-E’ reported in the original publication [45] was also found altered in the comparison between Tumor and NAT, which suggested that the altered proteins in the EMT pathway could be further investigated as potential biomarkers for diagnosis as well as prognosis.

## Conclusion

OmicsOne integrated multiple essential modules for phenotype association analysis and provided a comprehensive analysis to discover interesting phenotype-associated features (e.g., genes, proteins, or peptides modified by PTMs) in minutes. The data analysis results are displayed in an interactive dashboard in real-time. We demonstrated the performance of OmicsOne using the published data sets of HGSOC and LSCC in this study and believe it will be an efficient bioinformatics solution for investigating and evaluating phenotype associations with individual features or interested feature sets to understand aberrant biological processes.

## Supplementary Information

**Additional file 1: Figure S1.** The differential expression analysis applied on Tumor and NAT comparison of the proteome data of LSCC. A PCA plot can separate the Tumor and NAT samples clearly. B Volcano plot of differentially expressed proteins in Tumor and NAT samples

**Additional file 2: Table S1.** The analysis results of OmicsOne applied on the proteome dataset of HGSOC

**Additional file 3: Table S2.** The analysis results of OmicsOne applied on the glycoproteome dataset of HGSOC

**Additional file 4: Table S3.** The analysis results of OmicsOne applied on the glycoproteome dataset of LSCC

## Data Availability

OmicsOne is free available on GitHub (https://github.com/huizhanglab-jhu/OmicsOne). The proteomic and glycoproteomic data of HGSOC and LSCC was previously published [44, 45] and released with OmicsOne as sample data.

## Abbreviations

GO: Gene ontology
PTM: Post-translational modification
mRNA: Messenger ribonucleic acid
TMT: Tandem-mass-tag
DIA: Data independent acquisition
CV: Coefficient of variation
PCA: Principal component analysis
ORA: Over-representation analysis
BH: Benjamini-Hochberg
ROC: Receiver operating characteristic curve
AUC: Area under the ROC curve
