# Supplementary figures and images for: OmicsOne: associate omics data with phenotypes in one-click
Source: Clin Proteomics. 2021 Dec 11;18:29. doi: 10.1186/s12014-021-09334-w (PMC8903648; doi:10.1186/s12014-021-09334-w)

## Slide 1
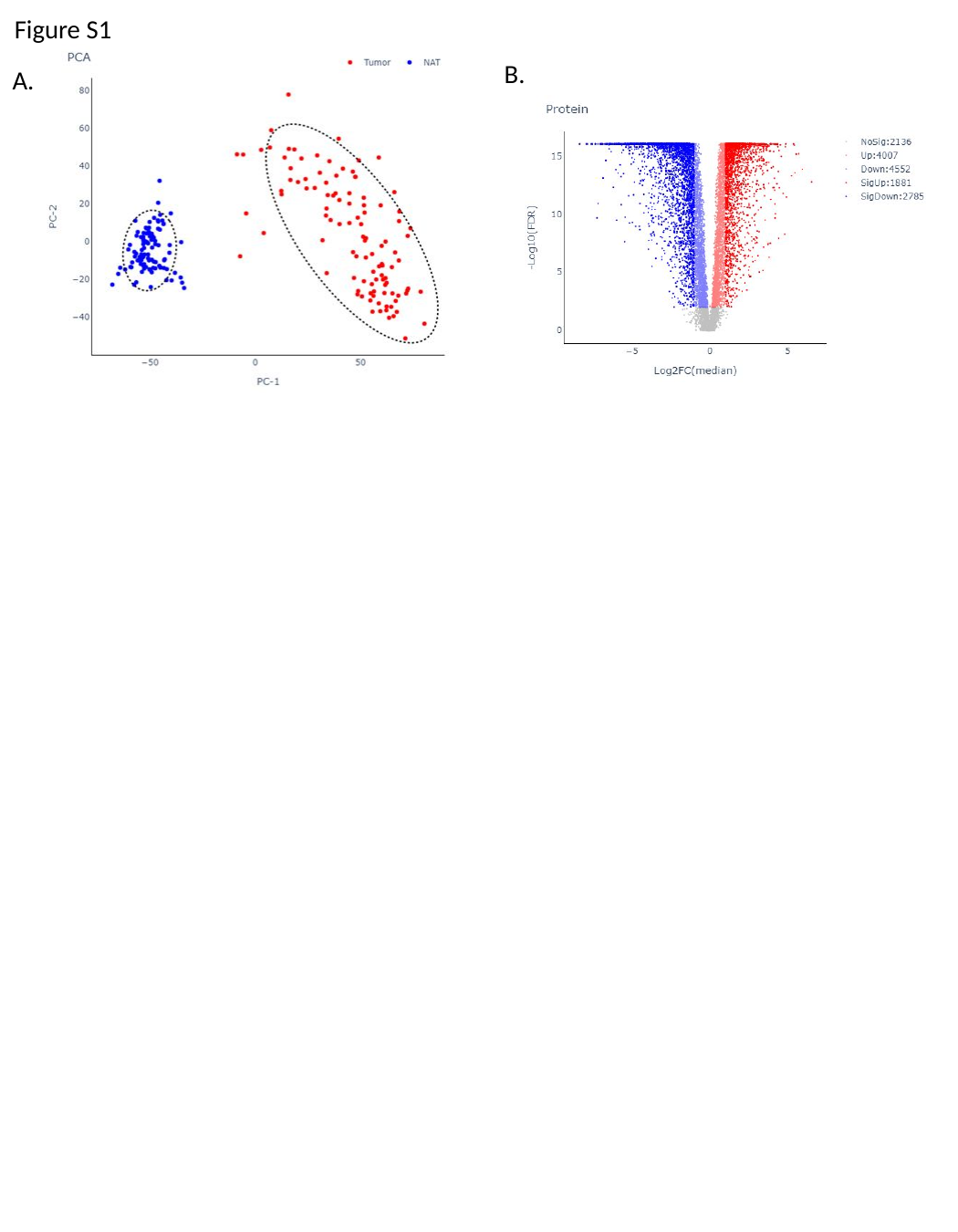

Figure S1
B.
A.

Supplement: Supplementary file 1 — Additional file 1: Figure S1. The differential expression analysis applied on Tumor and NAT comparison of the proteome data of LSCC. A PCA plot can separate the Tumor and NAT samples clearly. B Volcano plot of differentially expressed proteins in Tumor and NAT samples [file 12014_2021_9334_MOESM1_ESM.pptx]
